# Association of *UGT1A1* Variants and Hyperbilirubinemia in Breast-Fed Full-Term Chinese Infants

**DOI:** 10.1371/journal.pone.0104251

**Published:** 2014-08-07

**Authors:** Youyou Zhou, San-nan Wang, Hong Li, Weifeng Zha, Xuli Wang, Yuanyuan Liu, Jian Sun, Qianqian Peng, Shilin Li, Ying Chen, Li Jin

**Affiliations:** 1 Institutes of Biomedical Sciences, State Key Laboratory of Genetic Engineering and MOE Key Laboratory of Contemporary Anthropology, School of Life Sciences, Fudan University, Shanghai, China; 2 Department of Neonatology, Nanjing Medical University Affiliated Suzhou Hospital, Suzhou, China; 3 Center for Reproduction and Genetics and Suzhou Maternal-Child Medical Center, Nanjing Medical University Affiliated Suzhou Hospital, Suzhou, China; Thomas Jefferson University, United States of America

## Abstract

A retrospective case control study of breast-fed full-term infants was carried out to determine whether variants in *Uridine Diphosphate Glucuronosyl Transferase 1A1* (*UGT1A1*) and *Heme Oxygenase-1* (*HMOX1*) were associated with neonatal hyperbilirubinemia. Eight genetic variants of *UGT1A1* and 3 genetic variants of *HMOX1* were genotyped in 170 hyperbilirubinemic newborns and 779 controls. Five significant associations with breast-fed hyperbilirubinemia were detected after adjusting for gender, birth season, birth weight, delivery mode, gestational age and False Discovery Rate (FDR) correction: the dominant effect of rs887829 (c-364t) (Odds Ratio (OR): 0.55; 95% Confidence Interval (CI): 0.34–0.89; *p* = 0.014), the additive effect of (TA)_n_ repeat (OR: 0.59; 95%CI: 0.38–0.91; *p* = 0.017), the dominant effect of rs4148323 (Gly71Arg, G211A) (OR: 2.02; 95%CI: 1.44–2.85; *p* = 5.0×10^−5^), the recessive effect of rs6717546 (g+914a) (OR: 0.30; 95%CI: 0.11–0.83; *p* = 0.021) and rs6719561 (t+2558c) (OR: 0.38; 95%CI: 0.20–0.75; *p* = 0.005). Neonates carrying the minor allele of rs887829 (TA)_n_ repeat had significantly lower peak bilirubin than wild types, while the minor allele carriers of rs4148323 had significantly higher peak bilirubin than wild types. No association was found in *HMOX1*. Our findings added to the understanding of the significance of *UGT1A1* in association with neonatal hyperbilirubinemia in East Asian population. Additional studies were required to investigate the mechanisms of the protective effects.

## Introduction

Neonatal jaundice is a physiological and generally benign phenomenon. However, excessively high bilirubin concentration may cause permanent neural damage in newborns, i.e., “chronic bilirubin encephalopathy” or kernicterus [1]. Genetic and environmental factors contribute to the development of hyperbilirubinemia and the impact of genetic variation on this condition is increasingly recognized [2]. In particular, variants in the rate limiting enzymes in the bilirubin metabolism pathway may be associated with hyperbilirubinemia.

Uridine Diphosphate Glucuronosyl Transferase 1A1 (UGT1A1) is the key enzyme for bilirubin conjugation while unconjugated bilirubin is the main cause of hyperbilirubinemia [3]. A (TA)_n_ repeat located in the promoter region of the gene, *UGT1A1*, is found to be associated with the transcriptional activity [4]. In Caucasian newborns, the minor allele homozygote of this variant significantly increases bilirubin concentration during the first 2 days of life [5]. In addition, when the minor allele homozygote or heterozygote combined with *Glucose-6-Posphate Dehydrogenase* (*G6PD*) deficiency, the incidence of hyperbilirubinemia is increased [6]. However, in East Asian populations, such as Japanese and Chinese, the minor allele of rs4148323 (Gly71Arg, G211A) in exon 1, but not (TA)_n_ repeat, is found to be associated with neonatal hyperbilirubinemia [7], [8], [9].

Heme Oxygenase-1 (HMOX1) is another key enzyme in bilirubin metabolism pathway. It catalyzes the first and the rate-controlling step of heme degradation and generates biliverdin that is converted into bilirubin via biliverdin reductase [3]. A (GT)_n_ dinucleotide repeat is located in the promoter region of *HMOX1*, and the repeat is associated with transcriptional activity [10]. However, little is known regarding the clinical impact of the *HMOX1* variants on neonatal hyperbilirubinemia.

Exclusive breastfeeding is defined as a risk factor for development of severe hyperbilirubinemia [11], but it is the reference normative standard for infant feeding and nutrition as well [12]. Since East Asian ethnicity is considered as a high risk factor [11], some studies have been carried out in Japan and China Taiwan to explore the genetic factors associated with breast-fed neonatal hyperbilirubinemia [13], [14], [15]. None was done in China mainland. Therefore, we performed an exploratory study on association of variants of the two genes and hyperbilirubinemia in breast-fed full-term Chinese infants.

## Materials and Methods

This study retrospectively enrolled infants born between February and October in 2008 from The Municipal Hospital in Suzhou, China. Eligible infants were exclusively breast-fed, had an estimated gestational age of 37 weeks or above, had a birth weight of 2500 grams or above, and had no major abnormalities.

Every morning, transcutaneous bilirubin (TcB) was measured on the forehead of each neonate before discharge. Five transcutaneous bilirubinometers, including 3 KONICA MINOLTA JM-103 and 2 local branded were randomly used by the neonatologists. The hospital has adopted a discharge policy of 72 hours or above for newborns delivered vaginally and 120 hours or above for newborns delivered by cesarean section. Once the infant was discharged, no TcB was measured any more. Peak TcB of each neonate before discharge was reviewed.

According to China guideline in *Practical Neonatology* [16] and *Practical Pediatrics* [17], neonates were diagnosed as hyperbilirubinemia when their maximum TcB exceeded 12.9 mg/dL (221 µmol/L) on day 3 or later before they were discharged. Once the infants developed high concentration of bilirubin before day 3 or the pathological cause of hyperbilirubinemia was confirmed,infants would be transferred to the Neonatal Unit and excluded from our study. Otherwise, infants diagnosed as hyperbilirubinemia would receive phototherapy and remain in our study as cases. Newborns whose peak TcB during birth hospitalization stay were less than or equal to 12.9 mg/dL served as controls.

Blood samples for genotyping were obtained from surplus filter papers which were kept at 4°C for about one year after routine newborn screening.

The study was approved by both Suzhou Municipal Hospital Reproductive Medicine Ethics Committee and the Ethics Committee of Institutes of Biomedical Sciences. Since the data were analyzed anonymously, the filter paper was obtained from a standard screening procedure, and the TcB measurement was a completely noninvasive routine clinical assessment, both committees approved a waiver of written consent.

Each filter paper was placed in an Eppendorf tube, fully soaked in 1×PBS (pH = 8.0), and incubated in boiled water for 5 minutes. After a quick spin, the supernatant was transferred to a new tube, washed with absolute ethanol, centrifuged at 13,000 rpm for 10 minutes, and the supernatant was discarded. After a second wash with 70% ethanol, the precipitate was air dried. After resuspension in distilled H_2_O, the DNA extract was kept at −20°C until further use.

A final set of 11 genetic variants was selected for genotyping based on (1) functional significance, (2) tagging Single Nucleotide Polymorphisms (SNP) located at least 5 kb upstream and 2 kb downstream of *HMOX1* (chr22: 34102087–34122194) and *UGT1A1* (chr2:234328658..234348684), respectively [HapMap Data Release #27 (phase II+III), Feb 09, on NCBI B36 assembly, dbSNP b126], selected from the HapMap Han Chinese (CHB) population based on r^2^>0.8 and minor allele frequency (MAF)>0.1; (3) either G/A or C/T SNP combination due to the limitation of the SNPstream (Beckman Coulter) platform. These variants were able to capture 7 of 9 (78%) polymorphisms of *HMOX1*, 18 of 22 (82%) polymorphisms of *UGT1A1*. Information of the variants and the genotyping assays are listed in Table 1.

**Table 1:** Genotyped variants and genotyping assays.

Variant	assay	pirmers	oligo(5′->3′)
*UGT1A1*			
rs887829*	SNPstream(GA)	GA3U	CAACAAGTGTTACCAGAGAGGAA
		GA3L	TACAGTTGTGTTCTTTTCTTTCTAAAAG
		GA3S	CGTGCCGCTCGTGATAGAATGTGAACAAGTTAGGCTTCTTTTCCA
(TA)_n_ *	GeneScan(duplex)	TA-F	FAM-CCTTTGTGGACTGACAGCTTTTT
		TA-R	TTGCTCCTGCCAGAGGTTCG
rs4148323*	SNPstream(GA)	GA6U	ACATGAAATAGTTGTCCTAGCAC
		GA6L	ATTATGCCCGAGACTAACAAAA
		GA6S	GGCTATGATTCGCAATGCTTTGACGCCTCGTTGTACATCAGAGAC
rs1018124	SNPstream(CT)	CT7U	AGAAATGTAAGACATAAATTCAGTGTTC
		CT7L	AGAAGTATCATTTTCTCTAAGAGACTCAA
		CT7S	AGGGTCTCTACGCTGACGATTTTTTGGAGAAATACTTCTATTTAA
rs6717546	SNPstream(GA)	GA5U	TTCATTGCGTGTGCATGC
		GA5L	GTGTATTTGCCACAATTGTAACTG
		GA5S	GCGGTAGGTTCCCGACATATGAGAAAAGAAAAATAACCAGTAATC
rs11563250	SNPstream(GA)	GA1U	ACACCCTCATTAGCACAAAGTACT
		GA1L	ATCAGCTTGAAAAGAAATACAATCTC
		GA1S	ACGCACGTCCACGGTGATTTGAAAGCCTGTCAGTTTGATAGGAGA
rs6719561	SNPstream(CT)	CT9U	TGGCTGGAACACATTCTGT
		CT8L	TTGTAAACAAGAGTGCCTCAGT
		CT9S	GACCTGGGTGTCGATACCTACTCTACATCCTTGAGGCTGTGCAGT
rs4663972	SNPstream(CT)	CT9U	TGGCTGGAACACATTCTGT
		CT8L	TTGTAAACAAGAGTGCCTCAGT
		CT8S	GTGATTCTGTACGTGTCGCCAGGGACCCCAAGTTGCCATGACCTC
*HMOX1*			
(GT)_n_ *	GeneScan(duplex)	GT-F	FAM-CTTTCTGGAACCTTCTGGGACG
		GT-R	GGGGTGGAGAGGAGCAGTCAT
rs9607267	SNPstream(CT)	CT10U	TGGGGTTCAGAATAGGCC
		CT10L	AACAGCTGAAATGAAAGTGCTT
		CT10S	AGATAGAGTCGATGCCAGCTCAGGAAGGAGAATTGTGCCCTGTAG
rs2071749	SNPstream(GA)	GA7U	TATCTGTAAAATAGGGATAATAATGGTACC
		GA7L	TACAACTGATTCTCATGTCCCA
		GA7S	AGGGTCTCTACGCTGACGATTCTTAGACTTATAAGGCTTGAGTGA

*These were (potential) functional variants from published (and unpublished) studies.

(TA)_n_ repeat and (GT)_n_ repeat were amplified by a duplex PCR whose forward primers were labeled with FAM respectively. The products were electrophoresed on a Genetics Analyzer 3730xl (Applied Biosystems, Foster City, USA) according to the manufacturer's manual. Nine SNPs were genotyped using a GenomeLab SNPstream Genotyping System (Beckman Coulter Inc., Fullerton, California, USA) following the manufacturer's standard procedure. To ensure the reliability and the reproducibility of the genotyping, about 3–5% samples were repeated for each assay. The overall genotyping call rate was 95%, and concordance rate was higher than 99%.

Hardy-Weinberg equilibrium (HWE) for the (GT)_n_ repeat of *HMOX1* was performed using an exact test [18]. HWE for other variants, including bi-allelic-grouped (TA)_n_ repeat of *UGT1A1* were examined by Fisher's exact test.

Haplotypes and their frequencies were inferred using PHASE version 2.1.1 software (www.stat.washington.edu/stephens/phase/download.html). The Haploview program (www.broad.mit.edu/haploview/haploview) was adopted to identify block structures, and to calculate |D′| and r^2^ to measure linkage disequilibrium (LD).

Continuous variables and categorical variables were compared by *t* test and Pearson's *χ* ^2^ test, respectively. Univariate logistic regression analysis was performed to compute unadjusted and adjusted ORs with 95% CIs. Peak TcB comparison according to genotypes were examined by *t* test and adjusted by covariance (ANOCVA). All analyses were performed with SPSS version 16.0 software (SPSS Inc, Chicago, IL, USA). A two-sided probability value of <0.05 was considered statistically.

To avoid assumptions regarding the models of inheritance, additive, dominant, and recessive models for each bi-allelic polymorphism were assessed by SNPstats (http://bioinfo.iconcologia.net/SNPstats). The best-fitting inheritance model for each polymorphism was selected based on the lowest Akaike information criteria (AIC) [19]. For multiple hypotheses test, False Discovery Rate (FDR) was applied.

## Results

Initially, we have enrolled 331 hyperbilirubinemic infants. After controlling the filter paper's quality for further DNA analysis, only 170 hyperbilirubinemia participants left. After controlling the DNA quality, seven hundred and seventy-nine control participants were chosen at random within matching criteria of gender, delivery mode and birth season. The characteristics of the participants are summarized in Table 2. Cases and controls were comparable in gender, delivery mode, birth season, birth weight and gestational age. Peak TcB before discharge was significantly higher in hyperbilirubinemic infants than controls. The highest peak TcB was 18.0 mg/dL and 12.9 mg/dL in hyperbilirubinemic and control groups, respectively.

**Table 2:** Characteristics of hyperbilirubinemic patients and controls.

Variable	Case	Control	*p*
	(n = 170)	(n = 779)	
Gender,n(%)			
Male	89(52.4%)	421(54.0%)	0.69
Delivery mode,n(%)			
Vaginal	86(50.6%)	412(52.9%)	0.76
Cesarean	74(43.5%)	330(42.4%)	
Forceps	10(5.9%)	37(4.7%)	
Birth season,n(%)			
Winter (Feb,Mar)	21(12.4%)	96(12.3%)	0.77
Spring and Autumn (Apr,May,Sep,Oct)	106(62.4%)	465(59.7%)	
Summer (Jun,Jul,Aug)	43(25.3%)	218(28.0%)	
Birth weight,gram (mean±standard deviation)*	3437.4±415.8	3423.8±435.1	0.72
Gestational age, day (mean±standard deviation)	276.7±8.5	277.5±8.4	0.28
Peak TcB, mg/dL (mean±standard deviation)	14.7±1.1	10.4±2.0	2.1×10^−143^

*Fifteen cases and 78 controls had missing data.

All hyperbilirubinemic infants' bilirubin values dropped to 12.9 mg/dL or even less after phototherapy. None received exchange transfusion, none developed encephalopathy.

The counts of genotypes at all variants are presented in Table 3. The average call rate was 95%. Among the 11 genotyped variants, rs1018124 (80%), rs4663972 (89%) and rs6717546 (94%)'s call rate was lower than 95%. None of the variants showed significant deviation from HWE, except for rs2071749 (*p* = 0.04).

**Table 3:** Variations in *UGT1A1* and *HMOX1* genes in breast-fed newborns with hyperbilirubinemic patients and control subjects.

Gene	Variant	Case					Control					*p*(genotype)	*p*(allele)	OR(95%CI)^a^	*p*(allele)^b^	OR(95%CI)^b^
		m/m	M/m	M/M	MAF	HWE *p*	m/m	M/m	M/M	MAF	HWE *p*					
*UGT1A1*	rs887829	2	20	141	0.074	0.2	8	162	597	0.116	0.49	**0.035**	**0.025**	**0.61(0.39–0.94)**	**0.027**	**0.61(0.39–0.94)**
	(TA)_n_	1	22	146	0.071	0.58	13	154	604	0.117	0.38	0.054	**0.014**	**0.58(0.37–0.90)**	**0.015** d	**0.58(0.37–0.90)**
	rs4148323	8	73	85	0.268	0.17	20	220	510	0.173	0.61	**2.0×10^−4^**	**7.0×10^−5^**	**1.75(1.32–2.31)**	**8.0×10^−5d^**	**1.75(1.32–2.31)**
	rs1018124	1	41	94	0.158	0.2	23	206	394	0.202	0.62	0.137	0.095	0.74(0.52–1.06)	0.095	
	rs6717546	4	65	91	0.228	0.07	58	318	357	0.296	0.29	**0.023**	**0.015**	**0.70(0.53–0.93)**	**0.015** d	**0.70(0.53–0.93)**
	rs11563250	3	36	128	0.126	0.73	19	203	533	0.16	1	0.287	0.12	0.76(0.53–1.08)	0.12	
	rs6719561	10	71	77	0.288	0.33	111	330	301	0.372	0.21	**0.01**	**0.005**	**0.68(0.52–0.89)**	**0.005** d	**0.68(0.52–0.89)**
	rs4663972	1	38	115	0.13	0.47	13	188	491	0.155	0.38	0.43	0.271	0.82(0.57–1.17)	0.271	
*HMOX1*	rs9607267	39	79	48	0.473	0.54	157	389	217	0.461	0.51	0.647	0.686	1.05(0.83–1.33)	0.631	
	rs2071749	15	63	84	0.287	0.56	42	314	384	0.269	0.04	0.212	0.507	1.09(0.84–1.43)	0.507	
	(GT)_n_ c	SS	41	group1		0.08	SS	176	group1		0.08					
		SM	60	(SS_SM)			SM	297	(SS_SM)			6-grouped:	L:0.940	1.02(0.68–1.51)	0.94	
		SL	19	group2			SL	80	group2			0.931	M:0.727	0.96(0.74–1.23)	0.727	
		MM	30	(SL_MM_			MM	141	(SL_MM_			2-grouped:	S:/	1	/	
		ML	16	ML_LL)			ML	65	ML_LL)			0.804				
		LL	1				LL	10								

m:minor allele; M:major allele; MAF:minor allele frequency; HWE *p*:Hardy-Weinberg equilibrium test *p* value; OR: odds ratio; CI: confidence interval.

a Provided that OR of M allele was 1.

b Adjusted for gestational age, birth season, delivery mode, birth weight and gender. OR was provided when *p* value was below 0.05.

c (GT)_n_ repeat was classified into 3 groups (S\M\L). Provided that OR of S allele was 1.

d Retained significance after the FDR adjustment (<0.05×4/11).

Additionally, we found 3 (TA)_8_ alleles in the (TA)_n_ repeat. Because of its low frequency and reported decreasing promoter activity with an increasing number of (TA)_n_ repeat [20], we merged (TA)_8_ into (TA)_7_ for further analysis. Therefore, (TA)_n_ repeat was considered as bi-allelic variant.

LD results are shown in Table 4. Strong pairwise LD was observed in several pairs (each |D′|>0.8) within *HMOX1* and *UGT1A1*. But higher r^2^ values (each r^2^>0.5) only existed between rs887829 and (TA)_n_ repeat (r^2^ = 0.864), rs6717546 and rs6719561 (r^2^ = 0.508) in *UGT1A1*. An LD block structure was observed within *HMOX1*, including rs9607267 and rs2071749 ((GT)_n_ repeat was not analyzed). No block was found in *UGT1A1*.

**Table 4:** Linkage Disequilibrium.

	|D′|								
r^2^	***HMOX1***	rs9607267	rs2071749						
	rs9607267		**0.976**						
	rs2071749	0.308							
	***UGT1A1***	rs887829	(TA)_n_	rs4148323	rs1018124	rs6717546	rs11563250	rs6719561	rs4663972
	rs887829		**0.937**	**1**	**1**	0.588	0.394	0.642	0.582
	(TA)_n_	**0.864**		**0.829**	0.686	0.608	0.663	0.641	0.472
	rs4148323	0.028	0.02		0.728	0.751	0.737	0.766	**0.925**
	rs1018124	0.029	0.014	0.032		0.701	0.547	0.696	0.285
	rs6717546	0.017	0.018	0.054	0.286		**0.936**	**0.852**	0.785
	rs11563250	0.003	0.01	0.023	0.216	0.377		0.789	**1**
	rs6719561	0.088	0.088	0.077	0.195	**0.508**	0.199		**0.92**
	rs4663972	0.007	0.005	0.035	0.058	0.259	0.031	0.239	

r^2^>0.5 was shown in bold; |D′|>0.8 was shown in bold.

**Figure 1:**
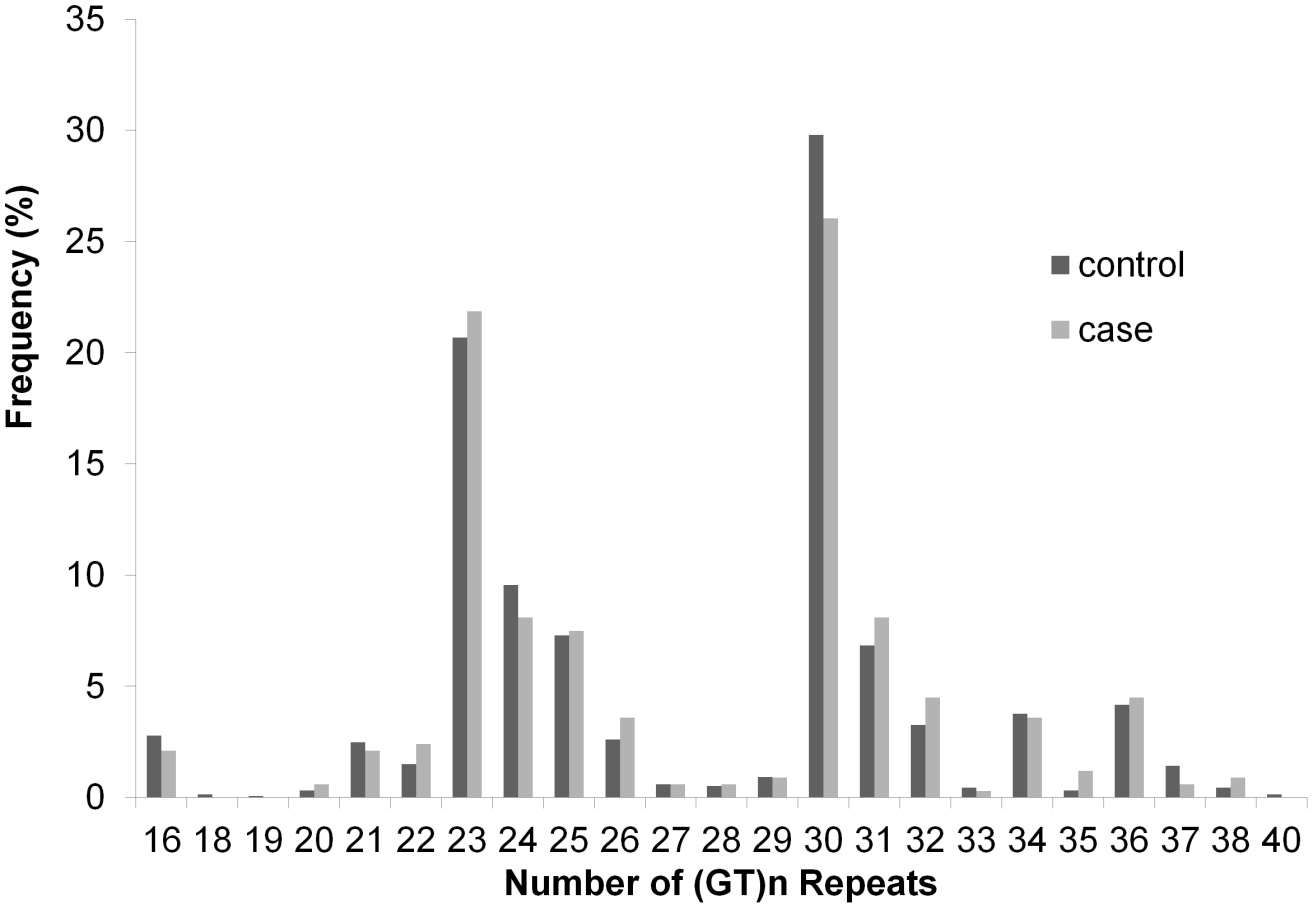
Distribution of (GT)_n_ repeat of *HMOX1*.

The distributions of (GT)_n_ repeat in *HMOX1* promoter in both case and control groups are shown in Figure 1. Both groups had four modes, located at 23, 30, 34 and 36, respectively. Using the classification defined by Yamada et al., the allele was classified into three classes according to the repeat length, namely, short alleles (S: <27 GT), middle alleles (M: 27–32 GT), and long alleles (L: ≥33 GT) [10]. The distributions of the genotypes are listed in Table 3. Neither genotypes nor allele distribution had significant difference between case and control groups. Based on previous publication, six genotypes could be merged into 2 groups: Group 1 (SS_SM) and Group 2 (SL_MM_ML_LL). Obviously, the *HMOX1* expression of Group 1 was expected to be higher than that of Group 2 [21]. However, we did not find an association between merged groups and neonatal hyperbilirubinemia (Table 3).

The associations of other variants are listed in Table 3. Distribution of genotypes of rs887829 (c-364t), rs4148323, rs6717546 (g+914a) and rs6719561 (t+2558c) in *UGT1A1* showed significant differences between case and control groups. Allele distributions of these variants, plus (TA)_n_ repeat, were associated with hyperbilirubinemia too. After controlling gender, birth season, birth weight, delivery mode and gestational age, the minor allele of rs4148323 was found to be a risk factor of hyperbilirubinemia (Odds Ratio (OR): 1.75; 95% Confidence Interval (CI): 1.32–2.31; *p* = 8.0×10^−5^); whereas, the minor alleles of the remaining variants were shown to be protective factors: rs887829 (OR: 0.61; 95%CI: 0.39–0.94; *p* = 0.027) and (TA)_n_ repeat (OR: 0.58; 95%CI: 0.37–0.90; *p* = 0.015) are two variants at the promoter region and are 310 nucleotides apart from each other; rs6717546 (OR: 0.70; 95%CI: 0.53–0.93; *p* = 0.015) and rs6719561 (OR: 0.68; 95%CI: 0.52–0.89; *p* = 0.005) are two tag SNPs located in 914 and 2558 nucleotides downstream of *UGT1A1*, respectively. After applying the FDR adjustment for multiple testing, only rs887829 lost statistically significance.

No variant in *HMOX1* was found to be associated with neonatal hyperbilirubinemia, neither was the LD block found in this gene (data not shown).

The result of best-fitting inheritance model analysis of associated variants was shown in Table 5. All of the five variants were associated with hyperbilirubinemia in its best-fitting inheritance model, and remained significantly after controlling gender, birth season, birth weight, delivery mode and gestational age and the FDR correction: the dominant effect of rs887829 (OR: 0.55; 95%CI: 0.34–0.89; *p* = 0.014), the additive effect of (TA)_n_ repeat (OR: 0.59; 95%CI: 0.38–0.91; *p* = 0.017), the dominant effect of rs4148323 (OR: 2.02; 95%CI: 1.44–2.85; *p* = 5.0×10^−5^), the recessive effect of rs6717546 (OR: 0.30; 95%CI: 0.11–0.83; *p* = 0.021) and rs6719561 (OR: 0.38; 95%CI: 0.20–0.75; *p* = 0.005) on neonatal jaundice.

**Table 5:** Estimated effects of selected variants on hyperbilirubinemia.

Variant	Best-fitting inheritance model	OR (95% CI)	*p*-value	OR (95% CI)^a^	*p*-value^a^
rs887829	Dominant	**0.55 (0.34–0.89)**	0.0097^b^	**0.55 (0.34–0.89)**	0.014^b^
(TA)n	Additive	**0.59 (0.38–0.91)**	0.011^b^	**0.59 (0.38–0.91)**	0.017^b^
rs4148323	Dominant	**2.03 (1.44–2.85)**	1×10^−4b^	**2.02 (1.44–2.85)**	5.0×10^−5b^
rs6717546	Recessive	**0.30 (0.11–0.83)**	0.0065^b^	**0.30 (0.11–0.83)**	0.021^b^
rs6719561	Recessive	**0.38 (0.20–0.75)**	0.0018^b^	**0.38 (0.20–0.75)**	0.005^b^

a Adjusted for gender, birth season, birth weight, delivery mode and gestational age.

b Retained significance after the FDR adjustment (<0.05×5/11).

LD test data shown in Table 4 demonstrated low r^2^ between rs4148323 and any of the aforementioned protective variants (each r^2^<0.08). Correspondingly, PHASE results also showed the frequency that the minor alleles of rs4148323 and rs887829 located on the same chromosome was 0.0019, as low as those of rs4148323 and (TA)_n_ repeat (frequency = 0.0018), rs4148323 and rs6717546 (frequency = 0.0188), and rs4148323 and rs6719561 (frequency = 0.0142). Furthermore, the frequency of all the minor alleles appearing on the same chromosome was 0. We speculated that the risk effect of the minor allele of rs4148323 and the protective effects of the minor alleles of the protective variants worked separately on the development of neonatal jaundice.

Comparisons of peak TcB according to *UGT1A1* genotypes were shown in Table 6. A general (additive) model was assumed for each polymorphism. The minor allele carriers (including both homozygous and heterozygous carriers) of rs4148323 had significant higher peak TcB than wild types (*p* = 2.9×10^−7^). On the other hand, the minor allele carriers of rs887829, (TA)_n_ repeat, rs6717546, or rs6719561 had significant lower peak TcB than wild types (*p* = 0.002, 0.001, 0.022 and 0.04, respectively). No difference was found in *HMOX1* genotypes. After applying the FDR adjustment, rs887829, (TA)_n_ and rs4148323 retained statistical significance.

**Table 6:** Comparison of peak TcB between different genotypes.

Gene	Variant		n	peak TcB, mg/dL	*p*	*p* a
				(mean±standard deviation)		
*UGT1A1*						
	rs887829	m/m+M/m	192	10.7±2.4	**0.002**	**0.002** b
		M/M	738	11.3±2.5		
	(TA)_n_	m/m+M/m	190	10.7±2.4	**0.001**	**0.001** b
		M/M	750	11.3±2.5		
	rs4148323	m/m+M/m	321	11.8±2.3	**2.4×10^−7^**	**2.9×10^−7b^**
		M/M	595	10.9±2.5		
	rs1018124	m/m+M/m	271	10.9±2.6	0.052	0.055
		M/M	488	11.3±2.5		
	rs6717546	m/m+M/m	445	11.0±2.6	**0.02**	**0.022**
		M/M	448	11.4±2.4		
	rs11563250	m/m+M/m	261	11.1±2.5	0.229	0.246
		M/M	661	11.3±2.5		
	rs6719561	m/m+M/m	522	11.1±2.5	**0.048**	**0.04**
		M/M	378	11.4±2.4		
	rs4663972	m/m+M/m	240	11.1±2.6	0.268	0.323
		M/M	606	11.3±2.5		
*HMOX1*						
	(GT)_n_	group1	574	11.2±2.5	0.893	0.77
		group2	362	11.2±2.5		
	rs9607267	m/m+M/m	664	11.2±2.5	0.753	0.934
		M/M	265	11.2±2.4		
	rs2071749	m/m+M/m	434	11.2±2.5	0.488	0.671
		M/M	468	11.3±2.5		

m:minor allele; M:major allele.

a Adjusted for gestational age, birth season, delivery mode, birth weight and gender.

b Retained significance after the FDR adjustment (<0.05×3/11).

## Discussion

Breastfeeding has been shown to have several advantages for infants, mothers, and families [12]. However, there is a strong association between breastfeeding and an increased risk of hyperbilirubinemia [11]. Since the etiology of breast-fed hyperbilirubinemia is unclear, associated factors should be systematically studied, especially in East Asian population, which is classified as a risk factor [11].

HMOX1 catalyzes the rate-limiting step in heme degradation. Based on the functional study, we suspected that shorter repeat carriers may have higher bilirubin level. Considering the distribution pattern of (GT)_n_ repeat, we tried more bi-allele definitions (S and L) other than the Yamada classification [10], including: <20 and ≥20, <23 and ≥23, <24 and ≥24 and <27 and ≥27, ≤32 and >32, ≤35 and >35. Unfortunately we did not find any association between (GT)_n_ repeat and hyperbilirubinemia. We hypothesized that the ‘short’ and ‘long’ cut-off value of (GT)_n_ repeat may be a grey zone instead of a single value.

The investigation of *UGT1A1* showed that rs4148323 (211G>A, Gly71Arg), located in the first exon, was a risk factor in neonatal hyperbilirubinemia, and was associated with higher peak TcB during hospitalization stay. Previous functional studies have shown that UGT1A1 enzyme activity of the minor allele carriers was significantly lower than that of major allele carriers [22], [23].

(TA)_n_ repeat in the promoter of *UGT1A1* is another extensively studied variant. Published functional studies have shown that the transcriptional activity of long repeat is significantly lower than that of short repeat [4], [20]. But the effect of long repeat on neonatal jaundice is not consistent. Risk effect has been observed in some Caucasian populations but not in Asian populations [5], [6], [7], [8], [24].Our study found the association between (TA)_n_ repeat and neonatal jaundice in an Asian population. Surprisingly, the long repeat appeared to be a protective factor and was associated with lower peak TcB during hospitalization stay, which was on the opposite of the functional study result.

Indeed, one recent association study between *UGT1A1* and neonatal jaundice in breast-fed Japanese newborns illustrated that heterozygous (TA)_7_ mutation significantly decreased the risk of hyperbilirubinemia (OR: 0.37; 95%CI: 0.15–0.89; *p* = 0.027) [15]. Another study in Taiwan showed the mean values of peak bilirubin in each (TA)_n_ genotype were significantly different: LL<SL<SS (*p* = 0.040) [14]. Furthermore, two meta-analysis studies also showed the protective effect of long repeat in two East Asian populations: Chinese and Japanese. Yang et al. found the frequency of (7/7+6/7) in control group was significantly higher than that of the hyperbilirubinemic group in both Chinese and Japanese newborns. The OR (95%CI) were 0.59 (0.36–0.96) and 0.15 (0.04–0.51), respectively [25]. Later, Long et al. calculated the OR (95%CI) of (7/7+6/7) as 0.02 (0.14–0.42), the OR (95%CI) of (TA)_7_ as 0.13 (0.04–0.42) [26].

In fact, it was found that longer (TA)_n_ repeat frequency was highest in African (49.5%), subsequently followed by European (38.7%) and Asian (16%) [20].And the hyperbilirubinemia guideline published by American Academy of Pediatrics (AAP) illustrated African origin was a protective factor whereas East Asian ethnicity was a risk factor for development of severe hyperbilirubinemia [11]. Taking these two opinions into consideration, we argued that (TA)_n_ longer repeat working as a protective factor for neonatal hyperbilirubinemia was more reasonable than a risk factor, although the mechanism (TA)_n_ longer repeat protect newborns from hyperbilirubinemia may be totally different from the known functional study. Due to the unexplained contradiction, further case control studies in East Asian population and functional analysis may help to disclose the mechanism.

Rs887829 (c-364t) is another variant located in the promoter of *UGT1A1*, 310 bp away from (TA)_n_ repeat. Strong LD between these two sites was found in our population (D′ = 0.937, r^2^ = 0.864), similar to the observation in a Japanese population and an African population respectively [27], [28]. There are no published functional studies on rs887829. One in vitro study showed that rs887829 did not affect the transcriptional activity of *UGT1A1* (unpublished data). We proposed that the protective effect of rs887829 may be due to the strong LD between this site and (TA)_n_ repeat.

Additionally, two tag SNPs, rs6717546 (g+914a) and rs6719561 (t+2558c), both located in the 3′ of *UGT1A1*, were associated with neonatal breast-fed hyperbilirubinemia too. So far there is no publication on these two sites. The only relative result we found was on rs4148329 (c+857t), which could be captured by rs6717546. That study showed significantly smaller bilirubin change before day 5 was observed for neonates who were homozygous for the minor allele versus those with wild-type (*p*<0.003) [29].In other words, rs4148329 minor allele carriers' bilirubin increased slowly so that they were at low risk for development of hyperbilirubinemia. That result of rs4148329 was consistent with ours on rs6717546. Unfortunately, no functional study was done on rs4148329. Therefore we proposed that the protective effects may be due to the strong LD with other potential functional sites.

This study has three limitations. (1) Due to short birth hospitalization stay, daily TcB measurement was normally stopped after day 4 for vaginal delivery and day 5 for cesarean delivery. We were not sure that some may become hyperbilirubinemia after discharge. But it is known that peak bilirubin normally reaches on day 4 and day 5, after that, the bilirubin either maintains or drops gradually [30], [31], [32]. Besides, short hospitalization stay is a common problem worldwide, hospitalization stay is even shorter in some foreign countries. Therefore, some published papers adopted data on day 3 or even before day 3 [13], [14]. (2) The diagnostic method our hospital used is different from AAP guidelines. Taking 12.9 mg/dL as the threshold from day 3 onwards is more stringent than the 95th centiles in Bhutani's nomogram from 72 hours onwards. This is probably due to the high risk of the ethnicity. (3) Number of cases is small. Although our case number was higher than two recently published breast-fed hyperbilirubinemia association studies [14], [15], and we recruited controls more than four times of cases to increase the statistical analysis power, the power of this study might be limited due to the small case number.

## Conclusions

Our study provides important new information on the association between genetic factors and breast-fed hyperbilirubinemia. Genotyping of *UGT1A1* may be a supplementary method to predict the development of hyperbilirubinemia in breast-fed full-term Chinese infants. Because of exploratory nature of our study and relatively limited number of examined subjects and variants, our conclusions should be validated in larger prospective trials. In addition, further studies were required to investigate the mechanisms of the protective effects of the variants.
